# The Sigma-1 Receptor: When Adaptive Regulation of Cell Electrical Activity Contributes to Stimulant Addiction and Cancer

**DOI:** 10.3389/fnins.2019.01186

**Published:** 2019-11-12

**Authors:** Olivier Soriani, Saïd Kourrich

**Affiliations:** ^1^Inserm, CNRS, iBV, Université Côte d’Azur, Nice, France; ^2^Département des Sciences Biologiques, Université du Québec à Montréal, Montréal, QC, Canada; ^3^Centre d’Excellence en Recherche sur les Maladies Orphelines – Fondation Courtois, Université du Québec à Montréal, Montréal, QC, Canada; ^4^Department of Psychiatry, University of Texas Southwestern Medical Center, Dallas, TX, United States; ^5^Department of Neuroscience, University of Texas Southwestern Medical Center, Dallas, TX, United States

**Keywords:** sigma-1 receptor, chaperone protein, voltage-gated ion channels, intrinsic excitability, plasticity, nervous system disorders, cancer, drug addiction

## Abstract

The sigma-1 receptor (σ1R) is an endoplasmic reticulum (ER)-resident chaperone protein that acts like an inter-organelle signaling modulator. Among its several functions such as ER lipid metabolisms/transports and indirect regulation of genes transcription, one of its most intriguing feature is the ability to regulate the function and trafficking of a variety of functional proteins. To date, and directly relevant to the present review, σ1R has been found to regulate both voltage-gated ion channels (VGICs) belonging to distinct superfamilies (i.e., sodium, Na^+^; potassium, K^+^; and calcium, Ca^2+^ channels) and non-voltage-gated ion channels. This regulatory function endows σ1R with a powerful capability to fine tune cells’ electrical activity and calcium homeostasis—a regulatory power that appears to favor cell survival in pathological contexts such as stroke or neurodegenerative diseases. In this review, we present the current state of knowledge on σ1R’s role in the regulation of cellular electrical activity, and how this seemingly adaptive function can shift cell homeostasis and contribute to the development of very distinct chronic pathologies such as psychostimulant abuse and tumor cell growth in cancers.

## Introduction

The sigma-1 receptor (σ1R, a.k.a., Sig-1R), also called sigma non-opioid intracellular receptor 1 and encoded by the gene *SIGMAR1* in humans, is actually not a receptor but a poorly characterized endoplasmic reticulum (ER) chaperone protein. Indeed, upon its discovery, σ1R was originally thought to be a novel opioid receptor (Martin et al., 1976). However, further pharmacological characterization shows that σ1R does not bind classical opioid ligands but rather binds (+)-benzomorphans (Walker et al., 1990; see review Schmidt and Kruse, 2019). σ1R has no known homologs in the eukaryotic genome and is the only known chaperone protein whose activity is regulated by endogenous and synthetic compounds in a clear agonist-antagonist manner (Hayashi and Su, 2007; Hayashi et al., 2011). At resting state, σ1R is coupled to BiP (Binding immunoglobulin Protein), another ER-resident chaperone protein. Upon agonist binding and in contrast to antagonist action, σ1R dissociates from BiP and acts as an interorganelle signaling modulator. Importantly, σ1R has been associated with many and diverse chronic diseases ranging from amyotrophic lateral sclerosis to Alzheimer’s disease, cancer and drug addiction (Romieu et al., 2004; Maurice and Su, 2009; Katz et al., 2011; Kourrich et al., 2012b; Yasui and Su, 2016). Among several distinct functions of σ1R that may contribute to these chronic pathologies, its ability to regulate and traffic a variety of functional proteins to the plasma membrane is gaining attraction (Su et al., 2010, 2016; Crottès et al., 2013; Kourrich, 2017). Specifically, σ1R regulates cellular electrical activity, a mechanism that occurs through physical protein-protein interactions with several VGICs and non-VGICs (Su et al., 2016; Kourrich, 2017; Schmidt and Kruse, 2019). In contrast to typical auxiliary ion channel subunits (e.g., Kvβs, Ca_*v*_β), this ability to associate and regulate functions and surface expression of a myriad of client ion channels from distinct superfamilies (i.e., sodium, Na^+^; potassium, K^+^; and calcium, Ca^2+^ channels) posits σ1R as an atypical auxiliary subunit (Aydar et al., 2002; reviewed in Kourrich, 2017). However, the use of *in vitro* model systems, such as cell culture models, *Xenopus* oocytes (Aydar et al., 2002; Crottès et al., 2011; Kinoshita et al., 2012) and neuroendocrinal tissue (Lupardus et al., 2000; Aydar et al., 2002) has been a major impediment to our understanding of how σ1R exerts its auxiliary subunit functions *in vivo.* Nonetheless, recent advancement provides evidence that σ1R can bind to ion channels in the brain and that endogenous or exogenous stimuli promotes the formation of these protein complexes, which results in long-lasting changes in cellular electrical activity (Crottès et al., 2013; Kourrich, 2017). The physiological and behavioral consequences of these changes are still largely unknown; however, with the exception of stimulant addiction and cancer, activation of σ1R are typically associated with both physiological and behavioral positive outcomes. Indeed, agonist activation of σ1R ameliorates the negative symptoms in many models of chronic diseases. For example, evidence suggests that activation of σ1R exhibits anti-amnesic effect against Aβ neurotoxicity in a model of Alzheimer’s disease (Jin et al., 2015); and while σ1R knockout (KO) mice exhibit a depression-like phenotype, σ1 agonists exhibit antidepressant properties (Hayashi et al., 2011; Wang et al., 2016). In stark contrast, activation of σ1R promotes the development of tumor growth in cancers (i.e., prostate, colorectal and breast cancers, and leukemia (Renaudo et al., 2007; Crottès et al., 2016; Gueguinou et al., 2017; Thomas et al., 2017) and addiction-relevant behaviors (Katz et al., 2016).

Stimulant addiction and cancer are very distinct types of severe chronic diseases. In both cases, many factors can trigger or contribute to their development and maintenance. These factors range from external stimuli such as intake or exposure to biological agents to internal factors such as genetic background that may confer vulnerability to the development of the disease. A common adaptation between stimulant addiction and some cancers is that both implicate enduring changes in cellular electrical activity, i.e., cellular intrinsic plasticity (Prevarskaya et al., 2010; Huang and Jan, 2014; Pardo and Stuhmer, 2014; Kourrich et al., 2015). The mechanism by which these changes are initiated and their contributions to the development and maintenance of the disease remain elusive. Nonetheless, recent studies revealed that σ1R is a common molecular player involved in the development of cellular electrical plasticity that characterizes both cancer (reviewed in Crottès et al., 2013) and psychostimulant drug addiction (Kourrich et al., 2013; Delint-Ramirez et al., 2018). We speculate that a reason that explains this opposite negative outcomes of σ1R activity on the development of cancer and psychostimulant abuse lies is the fundamental role of σ1R in cell’s health and survival, and especially, its ability to regulate cellular electrical properties.

As such, in this review, we will present the state of knowledge on σ1R-dependent regulation of ion channels. For the sake of brevity, we will focus on channels that directly contribute to the intrinsic electrical properties of the cell, in both neuronal and non-neuronal cells. Then, we will speculate on how this ability for σ1R to regulate cellular electrical activity, a seemingly adaptive function, can be hijacked and lead to chronic pathological conditions such as cancer and stimulant addiction.

## σ1R-Dependent Regulation of Ion Channels

### σ1R Is an Atypical Auxiliary Subunit for Ion Channels

σ1R has been proposed to be considered a ligand-regulated auxiliary potassium channel subunit since early 2000s (Aydar et al., 2002). However, whether σ1R is a *stricto sensu* auxiliary subunit is unclear, as the concept of auxiliary subunits has not been clearly defined. Auxiliary subunits are non-conducting, modulatory components of the multi-protein ion channel complexes that underlie normal neuronal signaling. Typically, auxiliary subunits, including σ1R, are characterized by several features, including (1) directly modulating the biophysical properties of the α pore-forming subunits (Aydar et al., 2002; Zhang et al., 2009; Kinoshita et al., 2012), (2) participating to the assembly, trafficking and surface expression of the pore-forming subunits (Crottès et al., 2011; Kinoshita et al., 2012; Kourrich et al., 2013; Balasuriya et al., 2014), (3) regulating ion currents in ligand-dependent and independent manner (Aydar et al., 2002; Kinoshita et al., 2012), (4) altering pharmacological interactions or bind drugs directly (see reviews Hayashi et al., 2011; Kourrich et al., 2012b; Schmidt and Kruse, 2019), and (5) interacting directly with the pore-forming subunit (Balasuriya et al., 2012, 2013, 2014). However, and in contrast to typical auxiliary subunits, indirect evidence suggest that σ1R is not stably associated with the pore-forming α subunits or present in purified channel complexes, but rather its association with client proteins is dynamic (Kourrich et al., 2013; Delint-Ramirez et al., 2018). Furthermore, σ1R exhibits specific functional characteristics that are not shared with typical auxiliary subunits. For example, canonical VGIC auxiliary subunits are either cytoplasmic proteins located at the plasmalemma level, i.e., in close proximity of the plasma membrane, or fully integrated in the phospholipidic membrane (Vacher et al., 2008). Instead, σ1R exhibits a heterogeneous distribution and can be found, in addition to these typical subcellular locations, at the nuclear and associated ER membranes, mitochondrial membrane (Hayashi and Su, 2003a, 2007; Luty et al., 2010; Su et al., 2010; Shioda et al., 2012) and potentially in extracellular space (Hayashi and Su, 2003a). Furthermore, while emerging evidence suggest that some channel family-specific auxiliary subunits have the ability to regulate ion channels from other superfamilies, such as the Na^+^ channel auxiliary subunits Na_*v*_β modulating voltage-gated K^+^ channels (Marionneau et al., 2012; Calhoun and Isom, 2014), this feature is extended and even exacerbated in σ1R. In particular, σ1R regulates via direct protein-protein interactions the functions of all VGIC superfamilies (i.e., Na^+^, Ca^2+^, and K^+^ families) and classes (i.e., ligand-gated ion channels, e.g., NMDA receptors, NMDARs; and G protein-coupled receptors, e.g., dopamine receptors, DARs) (reviewed in Crottès et al., 2013; Kourrich, 2017; Soriani and Rapetti-Mauss, 2017; Schmidt and Kruse, 2019). This unique characteristic positions σ1R as a powerful regulator of various cellular functions and neuronal excitability.

### σ1R-Dependent Regulation of VGICs

A feature that differentiates neuronal from non-neuronal cells is their excitability and their unique shape, which together endow neurons with the capability to receive, integrate and propagate the information within brain circuits. As such, this section will refer mainly to voltage-gated Na^+^, Ca^2+^, and K^+^ channels, i.e., the non-synaptic factors that are responsible for the generation and propagation of action potentials (Hille, 2001). This process is made possible thanks to a tight control of ion channels’ maturation, their delivery at the plasma membrane and their functional regulation. While the role of common K^+^ channel auxiliary subunits (e.g., Kvβs and KChips) is these processes are extensively studied (Maffie and Rudy, 2008; Vacher et al., 2008), the regulatory power of σ1R, emerging as a new but atypical auxiliary subunit (Aydar et al., 2002; reviewed in Kourrich, 2017), is scarcely understood. On that matter, we know today that σ1R regulates neuronal intrinsic excitability via trafficking of VGICs (particularly Kv channels) to the plasma membrane (reviewed in Kourrich, 2017), a function that operate in ligand-dependent and -independent manner. Though σ1R regulates the trafficking of VGICs from the ER to the cell surface, the mechanism remains unclear. Interestingly, σ1R possesses a double-arginine ER retention signal (RR) at the N-terminus (Hayashi and Su, 2003b). Because arginine-based intracellular retention signals are used to regulate assembly and surface transport of several multimeric complexes including ion channels (Scott et al., 2003; Gassmann et al., 2005; Phartiyal et al., 2008), it is tempting to speculate that such a mechanism may also apply to the regulation of VGICs by σ1R.

Modulation of neuronal intrinsic excitability is a critical mechanism that controls a plethora of physiological and cognitive functions. These functions range from the generation and propagation of information within brain circuits to the control of the capability of neurons to undergo synaptic plasticity, and thereby lead to lasting changes in behavior such as learning and memory. Yet, the regulatory power of σ1R remains elusive, partly because σ1R’s regulatory functions have been investigated using mainly heterologous cell culture systems (Aydar et al., 2002; Crottès et al., 2011; Kinoshita et al., 2012; reviewed in Kourrich, 2017). Further, the use of promiscuous ligands when investigating the regulatory functions of σ1R has so far prevented reaching molecular specificity.

**FIGURE 1:**
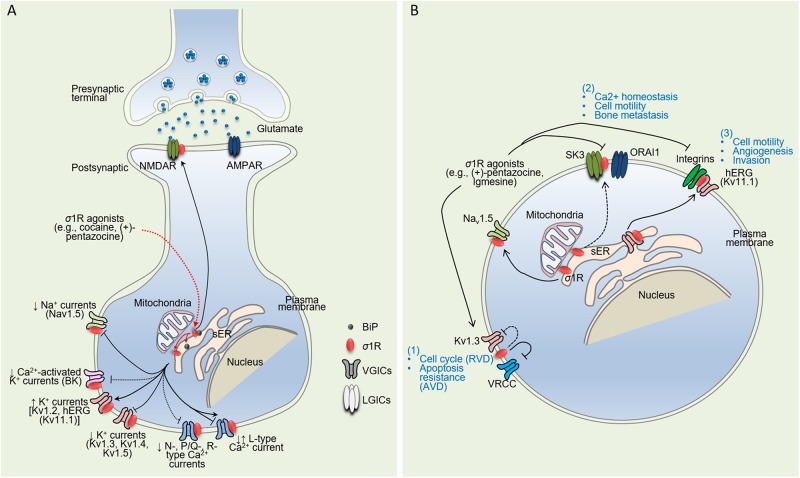
Schematic diagram illustrating direct σ1R-dependent regulation of ion channels in neuronal and cancer cells. **(A)** Upon ligand stimulation [e.g., cocaine, (+)-pentazocine, PRE-084], σ1R dissociates from binding immunoglobulin protein (BiP), another endoplasmic reticulum (ER) chaperone protein, and then translocates from the mitochondrion-associated ER membrane (MAM, interface between mitochondrion and ER) to the ER and plasmalemma. Acting as an interorganelle signaling modulator, σ1R regulates a variety of functional proteins, both directly and indirectly. Here are represented only the regulations mediated by direct interaction with the targets. Pointed and flathead arrows indicate positive and negative regulations respectively. Unbroken and dashed lines indicate direct and indirect evidence for σ1R:Ion channels physical interactions. On the one hand, σ1R upregulates ion channel expression at the plasma membrane either through the regulation of subunit trafficking activity (hERG) (Crottès et al., 2011) or a mechanism that remains unidentified (Kv1.2) (Kourrich et al., 2013; Delint-Ramirez et al., 2018). σ1R activation by (+)-SKF 10,047 enhances binding with NMDARs, a mechanism that may play a role in NMDAR subunits trafficking to the cell surface (Balasuriya et al., 2013; Pabba et al., 2014). On the other hand, σ1R inhibits ion currents through modulation of target’s biophysical properties (Kv1.3, Kv1.4) (Aydar et al., 2002; Kinoshita et al., 2012) and likely trafficking mechanisms (Na_*v*_1.5) (Johannessen et al., 2009; Balasuriya et al., 2012). This can occur through both ligand-independent (Kv1.3, Kv1.4, Kv1.5) (Aydar et al., 2002; Kinoshita et al., 2012) and ligand-dependent mechanisms (Kv1.4, Kv1.5) (Aydar et al., 2002). σ1R can both enhance (Sabeti et al., 2007) and inhibit (Tchedre et al., 2008) L-type Ca^2+^ currents. Adapted from Kourrich (2017). **(B)** By shaping cancer cell electrical signature, σ1R participates to cancer hallmarks. (1) σ1R functionally modulates VRCC and K^+^ channels restricting cell sensitivity to AVD without altering cell cycle progression; (2) σ1R binds SK3 channel and promotes the formation of SK3:ORAI1 complexes within cholesterol-rich nanodomains responsible for increased Ca^2+^ influx and migration potency; and (3) σ1R dynamically associates hERG channels to integrins upon cell stimulation by ECM triggering motility, angiogenesis and invasiveness.

σ1R-dependent regulation of VGICs can be mediated through direct protein-protein interactions or indirectly through G proteins. As such, σ1R has the capability to regulate neuronal intrinsic excitability and exert a strong influence on the ability for a neuron to generate action potentials in response to synaptic inputs, fine tune firing frequency, and conduct action potentials along the axon. This section summarizes evidence supporting σ1R regulations of VGICs and its resulting effects on their functions (Table 1 and Figure 1), which appear to be influenced by several factors, including the physiological milieu, brain regions, experimental preparations and neuronal types. However, a critical factor that exerts a strong influence on functional outcomes is σ1R’s conformational and oligomerization states, which appear to be dependent on the class of ligands used (Gromek et al., 2014; Mishra et al., 2015; reviewed in Chu and Ruoho, 2016).

**TABLE 1:** Summary of σ1R activation effects on VGICs.

**Functional effects on currents^∗^**	**Mode of activation**	**Experimental system**	**Mechanism/Evidence**	**References**
**Ca^2+^ currents**				

↓N, L, P/Q and R-type	Haloperidol, ibogaine, (+)-pentazocine, DTG	Parasympathic intracardiac neurons; superior cervical ganglia (cell culture)	Direct/2nd messenger systems and G proteins not required	Zhang and Cuevas, 2002^∗∗^
↓L-type	(+)-SKF10047	Retinal ganglion cells (cell culture)	Direct/co-IP	Tchedre et al., 2008

**Na^+^ currents**				

↓Nav1.5	(+)-pentazocine, (+)-SKF10047	Cell lines (HEK293, COS-7), Cardiac myocytes	Direct/2nd messenger systems and G proteins not required	Johannessen et al., 2009; Fontanilla et al., 2009
	None	tsA 201 cells, breast cancer cell lines (MDA-MB-231)	AFM	Balasuriya et al., 2012
	(+)-pentazocine	intracardiac ganglion neurons (isolated neurons from neonatal rats)	nd	Zhang et al., 2009

**K^+^ currents**				

↓*I*_*A*_	Pentazocine, SKF10047	Neurohypophysial terminals (pituitary gland slices)	Direct/2nd messenger systems and G proteins not required	Lupardus et al., 2000
	JO 1784, (+)-pentazocine	Neuroendocrine pituitary cell culture	Indirect/Gs protein required	Soriani et al., 1999a
↓*I*_*A*_ (Kv1.4)	SKF10047	*Xenopus* oocytes; rat posterior pituitary gland	Direct/co-IP	Aydar et al., 2002
↓*I*_*K(DR)*,_ *I*_*BK*_	DTG, (+)-pentazocine, ibogaine,	Parasympathic intracardiac neurons (cell culture), neuroendocrine pituitary cell culture	Direct/2nd messenger systems and G proteins not required	Soriani et al., 1998; Zhang and Cuevas, 2005
↓*I*_*M*_	(+)-pentazocine	Primary frog neuroendocrine pituitary cell culture	Indirect/Gs protein required	Soriani et al., 1999b
↑Kv1.2	*In vitro* and *in vivo* cocaine	Brain tissue (NAc and PFC); cell lines (NG108-15, Neuro2A, HEK293T)	Direct/co-IP	Kourrich et al., 2013; Delint-Ramirez et al., 2018
↓Kv1.3	±σ1R expression	*Xenopus* oocytes; HEK 293 cells	Direct/co-IP	Kinoshita et al., 2012
	(+)-pentazocine, igmesine, DTG	Jurkat cells	nd	Renaudo et al., 2004
↓Kv1.5	SKF10047	*Xenopus* oocytes	G protein-independent	Aydar et al., 2002
↑Kv2.1	Cyproheptadine, PRE-084	Mouse cortical neurons, HEK293T cells	Gi protein-dependent	He et al., 2012
↓*I*_*hERG*_ (Kv11.1)	Igmesine	*Xenopus* oocytes; HEK293; human K562; myeloid leukemia cells	Direct/co-IP	Crottès et al., 2011
	none	HCT116 human; colorectal cancer cells	Direct/FRET/Proximity Ligation Assay	Balasuriya et al., 2014
		HEK293	Direct/FRET, AFM	
↓Kir2.1	SKF10047	Mouse embryonic fibroblasts	nd	Wong et al., 2016

*^∗^All effects on ion channels’ current were obtained with electrophysiological recordings. ^∗∗^Rank order potency of various σR ligands suggest these effects may be through σ2R. AFM, atomic force microscopy; co-IP, coimmunoprecipitation; *I*_*A*_, A-type K^+^ current; *I*_*BK*_, large-conductance Ca^2 +^-activated K^+^ current; *I*_*hERG*_, human ether-à-gogo K^+^ current; *I*_*K(DR)*_, delayed outwardly rectifying K^+^ current; NAc, nucleus accumbens; nd, not determined; PFC, prefrontal cortex.*

#### Voltage-Gated Ca^2+^ Channels

Ca^2+^ influx through voltage-gated Ca^2+^ channels at the synapse play a key role in both fast synaptic transmission, i.e., allows the initiation of a mechanism necessary for neurotransmitter release, and in the regulation of intracellular signaling, thereby initiating slow and lasting changes in neuronal activity (Dolphin, 2009; Catterall, 2010). σ1R modulates intracellular Ca^2+^ concentration through both the regulation of membrane voltage-gated Ca^2+^ channels (Table 1 and Figure 1A) and Ca^2+^ mobilization from endoplasmic stores (Hayashi et al., 2000; Hayashi and Su, 2001; for reviews see Maurice and Su, 2009; Fishback et al., 2010; Su et al., 2010).

Although the subtype of σ receptors, σ1R or σ2R, was not identified yet, early indirect evidence linked σ receptors to modulations of Ca^2+^ channels’ functions (Rothman et al., 1991; Church and Fletcher, 1995; Brent et al., 1997). These studies, using binding, pharmacological and electrophysiological assays, showed that various σ receptor ligands inhibit intracellular Ca^2+^ dynamics. Although high concentrations of σ receptors’ ligands used in these studies did not exclude unspecific off-target effects, such as direct actions of ligands on Ca^2+^ channels rather than on σ receptors, the discovery of highly selective toxins for specific voltage-gated Ca^2+^ channels provided evidence that σ receptors associate with and regulate voltage-gated Ca^2+^ channels (Brent et al., 1997). Consistent with these findings, Cuevas and colleagues showed that σ receptor agonists, although the rank order potency of these ligands suggests these effects may be through σ2 rather than σ1 receptors, alters several Ca^2+^ channels’ biophysical properties on intracardiac ganglionic neurons (N-, L-, P/Q-, and R-type Ca^2+^ channels) (Table 1 and Figure 1A), all consistent with inhibition of Ca^2+^ influx. Interestingly, the mechanism through which σ receptors agonists exerted their inhibitory effects were independent on second messenger systems and G proteins (Zhang and Cuevas, 2002), suggesting that the putative σ receptor exerts its inhibitory action through protein-protein interactions with its client ion channel. To date, only the L-type voltage-gated Ca^2+^ channel has been identified as a direct target for σ1R (Tchedre et al., 2008). In particular, using both specific σ1R receptor ligands and co-immunoprecipitation assays, Tchedre et al. (2008) showed that σ1R activation with (+)-SKF 10,047 inhibits Ca^2+^ currents—an effect that is prevented by σ1R antagonist BD 1047 and that appears to be mediated via direct σ1R binding to L-type voltage-gated Ca^2+^ channel (Tchedre et al., 2008) (Table 1 and Figure 1A). In contrast, pregnenolone sulfate activation of σ1R increases L-type Ca^2+^ currents in the CA1 region of the hippocampus—a mechanism that promotes the induction of synaptic plasticity (i.e., long-term potentiation) (Sabeti et al., 2007). These findings suggest that the effects of σ1R ligands differ between preparations and/or regions of the nervous system. These discrepancies may also be due to the presence and type of ligands, which control σ1R’s conformational and oligomerization states (monomeric vs. oligomeric) (Gromek et al., 2014; Mishra et al., 2015; reviewed in Chu and Ruoho, 2016).

Besides voltage-gated Ca^2+^channels, σ1R also regulates non-voltage-gated Ca^2+^-permeable channels via protein-protein interactions, including IP3 receptors at the ER level (Hayashi and Su, 2007; reviewed in Maurice and Su, 2009; Fishback et al., 2010; Su et al., 2010), and plasma membrane acid-sensing ion channels 1a (ASIC1a) (Herrera et al., 2008; Carnally et al., 2010).

#### Voltage-Gated Na^+^ Channels

To date, and in contrast to bidirectional action of σ1R on Ca^2+^ currents, σ1R ligands exert inhibitory actions on Na^+^ currents (Table 1 and Figures 1A,B). Zhang et al. (2009) reported that σ1R ligand activation decreases neuronal intrinsic excitability, an effect mediated by hyperpolarization of Na^+^ channels’ steady-state inactivation and characterized by a decreased ability to initiate neuronal firing. In contrast, other studies found that σ1R ligand activation in various cell preparations [e.g., mouse cardiac myocytes, COS-7 and human embryonic kidney cells (HEK293)] inhibits Na_*v*_1.5-mediated currents without altering channel’s biophysical properties (Fontanilla et al., 2009; Johannessen et al., 2009). Since these effects were mediated in the absence of GTP or ATP, this mechanism is thought to be independent of G-proteins and protein kinases (Johannessen et al., 2009), suggesting direct interaction between σ1R and Na_*v*_1.5. Indeed, studies using atomic force microscopy (AFM) imaging provided direct evidence for σ1R-Na_*v*_1.5 protein-protein association (Balasuriya et al., 2012). However, since only 6% of the two proteins appears to interact, σ1R may be involved in Na_*v*_1.5 trafficking or maturation, a process that requires association-dissociation with Na_*v*_1.5. Strikingly, these authors also observed that the molecular silencing of σ1R in a breast cancer line reduced Na_*v*_1.5 current density and that (+)-pentazocine reduced interaction between σ1R and Na_*v*_1.5 alpha subunits (Balasuriya et al., 2012). Taken together, these results suggest that σ1R increases the number of channels at the plasma membrane, while σ1R ligands exerts inhibitory action through either decreasing Na^+^ current or rendering Na^+^ channels unavailable.

#### Voltage-Gated K^+^ Channels

Early seminal studies found that the σ1R modulates K^+^ currents (Bartschat and Blaustein, 1988; Kennedy and Henderson, 1990; Wu et al., 1991). However, results from Morio et al. (1994) were likely the first to suggest that this modulatory ability may involve direct interactions with K^+^ channels. Specifically, authors showed that σ1R ligand activation inhibits outward K^+^ conductance in GTP- and second messenger systems-independent manner (e.g., protein kinase A, PKA; and protein kinase C, PKC) (Morio et al., 1994). Later studies conducted in neuronal cells [i.e., rodent neurohypophysial nerve terminals (Lupardus et al., 2000) and parasympathic intracardiac neurons (Zhang and Cuevas, 2005)] provided additional evidence that σ1R agonist activation inhibits various K^+^ currents independently of ATP- and GTP-dependent processes, including delayed outward rectifier K^+^ current (*I*_*KDR*_), large conductance Ca^2+^-sensitive K^+^ channels (*I*_*BK*_) and M-current (Table 1 and Figure 1A). By contrast, in frog pituitary melanotroph cells (neuroendocrine cells), sigma ligands including Igmesine and (+)-pentazocine, increase action potential firing through a Gs-dependent inhibition of voltage-dependent K^+^ currents. Indeed, sigma ligands decrease *I*_*KDR*_ current density and accelerate *I*_*A*_ inactivation, together with a leftward shift in voltage-dependent inactivation curve (Soriani et al., 1998, 1999a). Moreover, (+)-pentazocine was shown to accelerate deactivation rate of the M-current, together with rightward shift in the steady-state activation curve (Soriani et al., 1999b) (Table 1).

To date, several K^+^ channels have been found to be regulated by direct σ1R binding (Table 1 and Figures 1A,B). Interestingly, this regulatory mechanism can occur through σ1R ligand-dependent or -independent mechanisms. Specifically, studies in *Xenopus* oocytes showed that in the absence of ligand, σ1R accelerates Kv1.4 inactivation time constant—an effect that is dependent on σ1R:Kv1.4 ratio—and similar to that observed with Kv1.3 (Kinoshita et al., 2012). Instead, application of the σ1R agonist SKF10,047 dramatically reduces outward K^+^ current of both Kv1.4 and Kv1.5 by ∼75% (Aydar et al., 2002). Using both *Xenopus* oocytes and neuroendocrine tissue (i.e., posterior pituitary gland), Aydar et al. (2002) found that this modulatory effect is mediated through σ1R-Kv1.4 protein-protein interactions, which is also further supported by co-localization studies in CHO-K1 cells (Mavlyutov and Ruoho, 2007).

Human Ether-a-go-go Related Gene (hERG, also named Kv11.1), another member of the voltage-dependent K^+^ channel family, also directly interacts with σ1R, as shown by co-immunoprecipitation, fluorescence resonance energy transfer (FRET) and AFM experiments (Crottès et al., 2011; Balasuriya et al., 2014). hERG is mainly expressed in the heart, brain or neuroendocrine tissues where it regulates firing pattern (Vandenberg et al., 2012). hERG is also aberrantly expressed in leukemias and epithelial cancers and has been clearly involved in disease progression (Becchetti et al., 2019). In HEK cells, σ1R increases maturation rate and channel stability at the plasma membrane. Accordingly, σ1R molecular silencing reduces current density in colorectal cancer (CRC) and leukemia cell lines, demonstrating that, in the absence of any ligand, σ1R stimulates hERG function (Crottès et al., 2011, 2013, 2016), a result which is in contrast with those obtained with Kv1.5 channels in *Xenopus* oocytes where σ1R behaves as a negative modulator (Aydar et al., 2002; Crottès et al., 2013). Noteworthy, cell incubation with the σ1R agonist igmesine (JO1784) produces the same effect as protein silencing on hERG current density. In contrast to Na_*v*_1.5/σ1R interaction, hERG/σ1R was resistant to (+)-pentazocine (Balasuriya et al., 2014). Interestingly, σ1R is required for the fast recruitment of hERG by integrins at the plasma membrane following cancer cell activation by the extra cellular matrix (ECM), suggesting that σ1R dynamically regulates ion channel activity depending on external stimulations (Crottès et al., 2016).

While most of previous studies were performed in heterologous systems (Aydar et al., 2002; Kinoshita et al., 2012) or in neuroendocrinal tissue *in vitro* (Aydar et al., 2002), σ1R-dependent modulation of Kv currents has also been shown to occur in the brain *in vivo* (Kourrich et al., 2013). Here, authors found that σ1R forms a complex with Kv1.2 channels in the nucleus accumbens (NAc), a critical hub of motivational neural networks, and the prefrontal cortex (PFC), a brain region involved in decisional processes. These protein complexes undergo enduring cocaine-driven upregulation in NAc inhibitory medium spiny projection neurons (MSNs) and thereby lead to persistent decrease in neuronal intrinsic excitability that lasts beyond the detoxification period—a neuroadaptation that contributes to behavioral response to cocaine (Kourrich et al., 2013). Cocaine-induced increase in σ1R-Kv1.2 complexes, a mechanism that promotes Kv1.2 trafficking to the plasma membrane, was also evidence in heterologous expression systems (NG108-15, Neuro2A and HEK293T cell lines), suggesting that the association between Kv1.2 and σ1R is a conserved mechanism (Kourrich et al., 2013; Delint-Ramirez et al., 2018). In sum, these studies demonstrate that the ability for σ1R to bind and regulate Kv functions and surface expression levels can be hijacked by external stimuli and lead to sustained maladaptive changes in cells’ electrical signature and contribute to nervous system disorders. Interestingly, a recent study confirming basal interaction between σ1R and Kv1.2 in HEK293T cells also found that this mechanism influences Kv1.2’s biophysical properties such as bimodal activation gating (Abraham et al., 2019).

Taken together, the σ1R associates with several K^+^ channels, and these associations can modulate K^+^ channels functions (Aydar et al., 2002; Kinoshita et al., 2012) or regulate subunits maturation and trafficking to the plasma membrane (Crottès et al., 2011; Kourrich et al., 2013; Balasuriya et al., 2014; Delint-Ramirez et al., 2018).

#### Non-voltage Gated Channels

σ1R not only binds voltage-gated channels. For example, AFM studies revealed that the chaperone also forms aggregates with Asic1a and NMDA receptors (Carnally et al., 2010; Balasuriya et al., 2013) and a recent work indicated a functional coupling with Kir2.1 channels (Wong et al., 2016). Last, in breast cancer cells, σ1R is tightly associated to SK3 (KCNN3) and promotes the functional association of the K^+^ channel to Orai1 Ca^2+^ channels. Interestingly, the coupling between the two channels is destroyed either in the absence of σ1R (molecular silencing) or by incubation with the sigma ligand igmesine (Gueguinou et al., 2017).

## σ1R:Ion Channels in Chronic Diseases: Stimulant Addiction and Cancer

σ1R is associated with distinct types of chronic diseases such as nervous system disorders, including Alzheimer’s disease, multiple sclerosis, depression, stimulant addiction, and cancer types such as colorectal, lung, prostate, breast cancers and leukemia (Renaudo et al., 2004, 2007; Romieu et al., 2004; Maurice and Su, 2009; Crottès et al., 2013; Kourrich et al., 2013; Aydar et al., 2016; Thomas et al., 2017). Nonetheless, the mechanism through which σ1R operates in such diseases remains elusive. Over the last few years, cancer research has accumulated evidence suggesting that σ1R, particularly *via* modulation of voltage-gated K^+^ channels, may contribute to various cellular functions that result in tumor growth (Crottès et al., 2013). Regarding stimulant addiction, another chronic disease, recent discoveries identified novel and unconventional σ1R-driven mechanisms that contribute to the development and likely the maintenance of psychostimulant abuse, some of which implicate enduring changes in neuronal intrinsic excitability (Delint-Ramirez et al., 2018). In addition to cancer and stimulant addiction, these findings extend the putative role for σ1R-driven intrinsic plasticity to other chronic neurological and neuropsychiatric disorders where both changes in intrinsic excitability and σ1R are engaged, e.g., Alzheimer’s disease, multiple sclerosis, and neuropathic pain. For the sake of brevity, this section will focus on stimulant addiction and cancer, two distinct type of chronic diseases whose contributing mechanisms implicate enduring changes in σ1R-driven trafficking of VGICs.

### Stimulant Addiction

Drugs of abuse, and especially psychostimulant drugs, enhance dopamine (DA) and/or other monoamines (e.g., serotonin, 5-HT; and noradrenaline, NA) signaling in brain reward circuits (Di Chiara and Imperato, 1988), including in the NAc shell and the prefrontal cortex. Overactivation of DA receptors triggers second messengers’ systems, involving protein kinases and phosphatases, leads to enduring changes in neuronal activity. To date, while the effects of psychostimulant drugs on glutamate synaptic strength has been extensively studied (Wolf, 2010, 2016; Luscher and Malenka, 2011; Wolf and Tseng, 2012), little is known about how they alter neuronal firing (Kourrich et al., 2015). While σ1R has long been associated with addiction to psychostimulant drugs [e.g., cocaine and methamphetamine (METH)] and alcohol (Romieu et al., 2004; Maurice and Su, 2009; Katz et al., 2011; Kourrich et al., 2012b; Yasui and Su, 2016), it is unclear whether and how σ1R contributes to these changes. Although several scenario have been proposed, a prominent hypothesis originates from σ1R’s ability to modulate monoaminergic systems, and thereby neuronal intrinsic excitability (Nichols and Nichols, 2008; Andrade, 2011; Ciranna and Catania, 2014). In this section, for the sake of brevity and due to the state of knowledge, we review evidence linking σ1R-driven plasticity of neuronal intrinsic excitability and addictive processes triggered by exposure to psychostimulant drugs; and when possible, we will discuss mechanisms by which monoamine signaling systems may also be involved.

Regarding psychostimulant drugs, although σR subtypes (σ1R vs. σ2R) were not identified yet, early studies from Ujike and colleagues found that σRs antagonists (BMY-14802, rimcazole or SR-31742A) block the development of psychomotor sensitization to cocaine and METH (Ujike et al., 1992a,b,c, 1996). Today, we know that this inhibitory effect of σR antagonists, and especially σ1R, extends to other addiction-relevant behaviors, including conditioned-place preference and drug self-administration, a model that closely mimic human condition (reviewed in Maurice and Su, 2009; Katz et al., 2011, 2016). While the conventional mechanism of action of psychostimulant drugs is to enhance DA (and other monoamines) signaling in the brain, accumulating evidence suggest that in addition to this mechanism, σ1R can exert its pro-addictive functions independently of DA signaling. An intriguing candidate mechanism originate from early studies that demonstrate that psychostimulant drugs such as cocaine and METH can directly activate σ1R in agonist-dependent manner (Sharkey et al., 1988; Kahoun and Ruoho, 1992; Nguyen et al., 2005; Chen et al., 2007; Hayashi and Su, 2007; Hayashi et al., 2011).

#### DA- and Monoamine-Independent Mechanisms

Rising evidence suggests that cocaine addiction also involves regional-specific alterations in intrinsic excitability (Nasif et al., 2005; Dong et al., 2006; Ishikawa et al., 2009; Kourrich and Thomas, 2009; for a review, see Kourrich et al., 2015). Specifically, it is well established that cocaine experience alters several VGICs, including Na^+^, Ca^2+^, and K^+^ conductances in NAc neurons (Zhang et al., 1998, 2002; Hu et al., 2004). Alterations of these channels are all consistent with decreased NAc MSNs intrinsic excitability (Dong et al., 2006; Ishikawa et al., 2009; Kourrich and Thomas, 2009; Mu et al., 2010). This adapatation is characterized by a decreased ability of neurons to trigger action potentials, and will be referred to as neuronal hypoactivity. However, evidence from both biophysical and pharmacological approaches found that the slowly inactivating A-type K^+^ current (also called D-type, *I*_*D*_) (Kourrich and Thomas, 2009; Kourrich et al., 2013) and the small conductance Ca^2+^-activated K^+^ current (SK type) (Ishikawa et al., 2009; Mu et al., 2010) play a significant role in the expression of cocaine-induced neuronal hypoactivity. Importantly, this neuroadaptation is observed after weeks of cocaine abstinence from both non-contingent intraperitoneal injection (Ishikawa et al., 2009; Kourrich and Thomas, 2009) and contingent intravenous cocaine self-administration (Segev et al., 2018; Wang et al., 2018).

Whereas enhanced DA signaling is accepted as the canonical mechanism initiating addictive processes, evidence indicates that cocaine also triggers non-canonical, DA-independent mechanism(s) that contribute to addiction (Sora et al., 1998), some of which involve σ1R (Garces-Ramirez et al., 2011; Katz et al., 2011; Hiranita et al., 2013; reviewed in Katz et al., 2011, 2016). These mechanisms are not excluding the critical role of DA in the development of stimulant addiction; however, unconventional DA-independent (or monoamine-independent) are still underestimated by the scientific community. Recent studies demonstrate that cocaine-induced neuronal hypoactivity in NAc shell MSNs is independent of DA, and potentially other monoamine signaling (Delint-Ramirez et al., 2018). In particular, upon both *in vivo* and *in vitro* cocaine exposure, σ1R associates with Kv1.2 channels, a channel that drives *I*_*D*_ in striatal MSNs (Shen et al., 2004). This mechanism promotes Kv1.2 trafficking from the ER to the plasma membrane, which enhances *I*_*D*_, decreases neuronal firing, and thereby enhances behavioral response to cocaine (Kourrich et al., 2013; Delint-Ramirez et al., 2018) (Figure 1A). Consistent with a DA-independent mechanism, cocaine-induced σ1R-Kv1.2 protein-protein associations and enhanced membrane Kv1.2 also occurs in HEK293T cells that are devoid of DARs. Although other voltage- and Ca2^+^-gated ion channels are thought to contribute to cocaine-induced neuronal hypoactivity (Zhang et al., 1998, 2002; Hu et al., 2004; Ishikawa et al., 2009) and that σ1R can modulate all classes of VGICs (Figure 1A), it is still unknown whether changes in these channels after exposure to cocaine implicate σ1R-dependent trafficking mechanisms.

While conventional mechanisms of actions for abused substances involve extracellular targets, DA transporters (DAT) (and other monoamine transporters) in the case of psychostimulant drugs, work form Henry Lester’s group provide an alternative hypothesis. Here, drugs of abuse or psychiatric drugs in physiological milieu coexist with their deprotonoted form (membrane permeant). These neutral forms can freely diffuse through the plasma membrane and act directly on intracellular targets. σ1R is enriched in intracellular organelles, especially at the ER level. Binding assays carried out on cell homogenates — a preparation that nevertheless does not preserve plasma membrane integrity — showed that cocaine, at doses found to be rewarding in rodents, binds to σ1R (Kahoun and Ruoho, 1992; Chen et al., 2007). Using a combination of *in vitro* molecular and electrophysiological approaches in HEK293T cells and NAc shell brain slices, respectively, Delint-Ramirez et al. (2018) demonstrated that cocaine binding to intracellular σ1R initiates the mechanism responsible for MSNs hypoactivity. Together, this suggests that beyond canonical DA-driven mechanisms initiated by extracellular actions of cocaine, cocaine also exerts its addictive properties *via* intracellular actions triggered by direct binding to σ1R (Garces-Ramirez et al., 2011; Hiranita et al., 2013; reviewed in Katz et al., 2016). This is consistent with the work form Dr. Katz group demonstrating that animals with cocaine experience self-administer σ1R agonists (e.g., PRE-084 and (+)-Pentazocine) (Hiranita et al., 2013) at doses that do not induce DA release in the NAc shell (Garces-Ramirez et al., 2011; reviewed in Katz et al., 2016). This switch in reinforcement does not occur after experience with food reinforcement (reviewed in Katz et al., 2016).

Importantly, METH, a psychostimulant drug that is structurally distinct from cocaine and that exhibits high addiction liability, also depresses NAc shell neuronal excitability (Graves et al., 2015), an effect that may be triggered by METH binding to σ1R (Nguyen et al., 2005). Indeed, structural and crystallography studies found that σ1R ligand-binding cavity is capable of binding structurally different compounds (Su et al., 2010; Schmidt et al., 2016).

#### Putative DA-Dependent Synergistic Mechanisms

In regards to the implication of σ1R in the development of addictive processes, σ1R and DA 1 receptors (D1Rs) can form heteromers. Cocaine binding to this complex amplifies D1R-mediated increases in cyclic AMP (cAMP), a mechanism that appears to occur in mouse striatum (Navarro et al., 2010). Although the complex formed by the D1R and σ1R appears to amplify PKA signaling, the mechanisms by which this adaptation contributes to cocaine addiction has not been identified yet. A putative mechanism may be the activation of PKA and thereby the inhibition of striatal *I*_*D*_ current, which is expected to enhance neuronal firing. Indeed, previous studies indicate that inhibiting PKA pathways decreases neuronal firing (Hopf et al., 2003; Perez et al., 2006), therefore excluding this scenario as a putative mechanism for cocaine-induced neuronal hypoactivity in NAc shell MSNs, a mechanism that occurs in D1R- but not in DA 2 receptors (D2R)-containing MSNs (Delint-Ramirez et al., 2018). An important consideration to take account is that although the complex formed by the D1R and the σ1R occurs, the ability for cocaine to activate this protein complex *in vivo* is yet to be demonstrated. Indeed, authors applied cocaine *in vitro* at 30–300 μM (Navarro et al., 2010), a concentration that exhibits off-target effects, especially on VGICs (Reith et al., 1986; Zimanyi et al., 1989; Wheeler et al., 1993; O’Leary and Chahine, 2002; Xiao et al., 2004; Chen et al., 2006). Furthermore, this concentration range is significantly beyond cocaine concentration found in the brain upon exposure to standard non-toxic rewarding dose (l–3 μM) (Pettit et al., 1990), and even higher than the total serum concentrations of cocaine (>10 μM) that produce toxicity in humans (Van Dyke et al., 1976; Paly et al., 1982; Escobedo et al., 1991). Nonetheless, assuming that σ1R-D1R complex plays a role in the behavioral effects of cocaine, the signaling pathway that is initiated does not seem to result in changes in intrinsic postsynaptic neuronal excitability. One possible mechanism by which this heteromeric complex may contribute to addictive processes may be by enhancing presynaptic glutamate release in prelimbic cortex (Dong et al., 2007), a region in brain reward circuits that is critical for initiating cocaine seeking and relapse to drug seeking behaviors (McFarland and Kalivas, 2001; Capriles et al., 2003; McFarland et al., 2003, 2004; McLaughlin and See, 2003; for reviews, see Kalivas and O’Brien, 2008; Peters et al., 2009).

An intriguing candidate mechanism that may also implicate DAR-activated kinases in the regulation of neuronal intrinsic excitability is the activation of the α-isoform of calcium/calmodulin-dependent protein kinase II (αCaMKII). Indeed, D1R activation enhances αCaMKII (CaMKII-pThr286) (Anderson et al., 2008), and besides its established role in glutamate AMPAR trafficking to the surface, αCaMKII regulates surface K^+^ channel density and currents directly, especially A-type K^+^ currents (Yao and Wu, 2001; Varga et al., 2004; Sergeant et al., 2005). A previous study found that mice that overexpress a constitutively active form of striatal neuron-specific αCaMKII (in which Thr286 is mutated to Asp) exhibit a decreased MSN firing capacity in the NAc shell in the absence of any detectable changes in glutamate transmission (Kourrich et al., 2012a). Interestingly, while σ1R also has the capability to increase αCaMKII-pThr286 (Moriguchi et al., 2011), whether this mechanism is downstream of D1R activation and results in modulation of neuronal firing is yet to be investigated.

#### Behavioral Relevance

From a behavioral viewpoint, neurobiological manipulations that promotes NAc neuronal hypoactivity (e.g., viral enhancement of K^+^ currents or via overexpression of αCaMKII-pThr286) enhances rewarding properties of cocaine (Kourrich et al., 2012a) and produce a hypersensitivity to cocaine’s psychomotor effects (Dong et al., 2006; Kourrich et al., 2012a)—a mechanism that is thought to contribute to the transition from recreational to compulsive drug use (Ferrario et al., 2005; Robinson and Berridge, 2008). At the circuit level, enhanced behavioral response to cocaine following inhibition of NAc shell neurons is thought to be driven by the disinhibition of downstream reward-related brain regions. In sum, the decreased neuronal intrinsic excitability in the NAc shell is an adaptation sufficient to promote addiction phenotype and is now considered as one of the hallmarks for cocaine addiction (reviewed in Kourrich et al., 2015). This adaptation is also consistent with the predominant tonic inhibition in NAc shell during short-access cocaine self-administration sessions (Peoples et al., 1998, 1999; Peoples and Cavanaugh, 2003) and the role of the NAc hypoactivity in cocaine behavioral effects (Peoples et al., 2007).

Together, accumulating evidence demonstrate that in addition to the established role of DA in initiating and shaping addiction-relevant behaviors, cocaine (and potentially other psychostimulant drugs) engages unidentified mechanisms that also contribute to shaping the addiction phenotype (Kuribara and Uchihashi, 1993; Mattingly et al., 1994; White et al., 1998; Prinssen et al., 2004). Accordingly, a recent review from David J. Nutt and colleagues discusses evidence that enhanced DA release in the brain may not be the sole mediator or initiator for addictive processes (Nutt et al., 2015). This underscores the high clinical significance for identifying novel mechanisms of actions for drugs of abuse, e.g., DA-independent, which would stimulate the development of novel and combinatorial pharmacotherapies to treat stimulant abuse or to provide alternatives for individuals resistant to conventional treatments.

In a larger extent, the discovery that σ1R can form complexes with specific VGICs and that these protein complexes can undergo enduring experience-dependent maladaptive plasticity suggests new mechanistic hypotheses for other neuropsychiatric disorders that are associated with alterations in σ1R and VGICs functions (e.g., Alzheimer’s’ disease and multiple sclerosis).

### Cancer

#### Ion Channels in Cancers

While the presence of ion channels has been observed for decades in cancer cell lines (Strobl et al., 1995), the understanding of their influence on cancer biology has only emerged recently. Instability in cancer cell genotypes induce several pathological features (hallmarks) including proliferation, resistance to apoptosis, epithelial to mesenchymal transition, tissue invasion and angiogenesis (Hanahan and Weinberg, 2011). Moreover, cancer cells entertain a dynamic bi-directional dialog with their local microenvironment, formed by cancer-activated fibroblasts, immune cell, blood vessels and proteins of the extra-cellular matrix, triggering signaling pathways shaping the development of the disease (Quail and Joyce, 2013). A general scheme has emerged in which ion channels shape an electrical signature that accompanies all steps of disease development, relapse and finally patient survival (Wulff et al., 2009; Pardo and Stuhmer, 2014; Arcangeli and Becchetti, 2017; Rapetti-Mauss et al., 2017; Prevarskaya et al., 2018; Becchetti et al., 2019).

#### σ1R in Cancers

The functional relationships between σ1R and ion channels in the central nervous system (CNS) raises the question of the function of the chaperone in cancers. In the 1990’s, several teams pointed out the presence of high densities of σ1R in cancer cell lines (i.e., glioblastoma, neuroblastoma, leukemia, breast cancer, melanoma, prostate or lung cancer cell lines) and suggested that it could be involved in cancer cell biology since sigma ligands (e.g., BD737) decreased cell proliferation or induced cell death *in vitro* (Vilner et al., 1995a, b). Despite the large number of publications dealing with σ1R and cancer, most of the knowledge until recently came from descriptive pharmacological studies with few answers regarding the innate function of the protein in disease progression as well as the molecular mechanisms involved. However, the evolution of the concept of σ1R from a classical receptor to a ligand-regulated chaperone protein has been accompanied by new information on its functions in cancer cell biology.

Spruce and collaborators showed that σ1R expression in breast and prostate cancer cells induced a break on Caspase-3-dependent apoptosis that could be attenuated by the σ1R antagonist rimcazole, in turn reducing tumor growth and metastatic proliferation in mice xenograft models. Accordingly, the effects of rimcazole could be attenuated by the σ1R agonists SKF10.047 and (+)-pentazocine (Spruce et al., 2004). In Jurkat T cell lines, it was observed that σ1R expression increased cell resistance to thapsigargin-induce apoptotic volume decrease (AVD), an early step in cell death process (Renaudo et al., 2007). In Jurkat and small cell lung cancer cells (NCI-H209), sigma ligands [igmesine and (+)-pentazocine] blocked cell cycle by inhibiting regulatory volume decrease (RVD), a mechanism related to AVD and that is required for G1/S transition (Renaudo et al., 2004). Overexpression of σ1R when compared to healthy tissues is also observed in colorectal (CRC) and myeloid leukemia (ML). In these cancers, σ1R promotes integrin-dependent invasive process *in vitro* and *in vivo*. In breast cancer patients, σ1R expression correlates with reduced overall survival; and at the cellular level, σ1R potentiates Ca^2+^-dependent migration, a mechanism also found in CRC cells (Crottès et al., 2016; Gueguinou et al., 2017). In prostate cancer, a recent study revealed that σ1R expression contributes to cell resistance to ER stress, and to the development of treatment-induced castration-resistant prostate cancer by promoting androgen receptor resurgence (Thomas et al., 2017).

Altogether, these results suggest that σ1R plays a significant role in cancer cell by regulating ion channel-related mechanisms (e.g., AVD, RVD, integrin signaling, Ca^2+^ homeostasis) (Aydar et al., 2016; Crottès et al., 2016; Guéguinou et al., 2016; Soriani and Rapetti-Mauss, 2017; Rapetti-Mauss et al., 2018).

##### Cancer cell growth and volume regulation

Cancer cell growth is the result of a balance between cell division and cell apoptosis. Early works by Bowen and coworkers back in the 1990s revealed that incubation of cancer cell lines with sigma ligands such as haloperidol and BD737 impaired cell growth *in vitro*. Interestingly, cell growth arrest was accompanied by a cellular swelling (Vilner et al., 1995a). Further works revealed that sigma ligands such as (+)-pentazocine or igmesine induced a cell swelling *in vitro* by inhibiting Kv and volume regulated chloride channels (VRAC). In fact, these two channels are the main effectors of the RVD process, allowing, in response to hypo-osmotic challenge, cell volume recovery by allowing potassium chloride (KCl) and osmotic water efflux (through activation of K^+^ and chloride channels). RVD is required for cells to pursue their cell cycle through the G1/S phase transition (Rouzaire-Dubois and Dubois, 1998; Rouzaire-Dubois et al., 2000). Accordingly, in SCLC and leukemia cell lines, inhibition of Kv and VRAC channels by σ1R ligands provoked accumulation of the p27kip1, and a decrease in cyclin A contents, reflecting an arrest of cell cycle at the end of the G1 phase (Renaudo et al., 2004, 2007). Cell volume regulation is also an important feature in the context of cell death. Indeed, cell shrinking by water efflux following activation of VRAC and K^+^ channels, is an early event of apoptosis signaling (Maeno et al., 2000; Lambert et al., 2008). In the studies performed by Renaudo and colleagues (2004, 2007), several clues indicated that σ1R was also involved in cell response to apoptosis. In leukemia T cells, the sigma ligand igmesine reduced VRAC current density and delayed staurosporine-induced AVD. Experiments in HEK293 cells showed that σ1R overexpression decreased volume-regulated chloride channels (VRCC) activation rate and delayed stausporine-induced AVD.

Taken together, these data suggest that σ1R expression in cancer provides cells better resistance capacity to apoptotic signals. Indeed, the tonic brake exerted by σ1R on VRAC channel activation rate restricts AVD without impeding RVD, the latter being necessary for cell cycle progression (Renaudo et al., 2007). This idea, in which σ1R exerts membrane-channel dependent pro-survival function seems to mirror the model proposed by Hayashi and Su in which σ1R, by chaperoning IP3R at the mitochondrion-associated ER membrane (MAM), stimulates CHO cell resistance to ER stress-induced apoptosis (Hayashi and Su, 2007).

##### Calcium influx and cell migration

In cancer, mortality is mainly the consequence of metastasis spreading, a complex process requiring the capacity of cells to migrate from the primary site and disseminate. Among the cellular processes involved in cell migration, Ca^2+^ homeostasis is crucial. Indeed, a deep Ca^2+^ influx remodeling occurs during the development of the disease and recent findings have revealed the formation of gain-of-function ion channel platforms at the plasma membrane as key events for metastasis progression (Guéguinou et al., 2014; Monteith et al., 2017). In breast and colorectal cancers (BC and CRC), σ1R is required to trigger the physical and functional coupling between the Ca^2+^ channel Orai1 and the Ca^2+^-dependent K^+^ channel SK3 (*KCNN3*). The group of Vandier has shown that the coupling of these two channels within cholesterol-rich membrane nanodomains (labeled by the presence of Caveolin1) potentiated constitutive (in BC) or capacitive (in CRC) Ca^2+^ influx, which in turn stimulated cell migration and bone metastasis formation in BC (Chantôme et al., 2013; Guéguinou et al., 2016). Co-immunoprecipitation and FRET assays demonstrated that σ1R binds SK3. Furthermore, σ1R silencing abrogated SK3-dependent Ca^2+^ influx and migration by chasing both SK3 and Orai1 out of caveolae lipid nanodomains. Interestingly, the sigma ligand igmesine mimicked these effects on Ca^2+^ influx and migration by dissociating Orai1 from SK3, the former being excluded from lipid caveolae nanodomains in MDA MB 435s BC cells. Noteworthy, σ1R is overexpressed in CRC and BC human samples and is associated with higher-grade tumors in CRC and reduced overall survival in BC patients (Gueguinou et al., 2017).

##### Na_*v*_1.5 channels and invasiveness

Voltage-gated sodium channels (VGSC) have been extensively characterized for their electrogenic functions in cell excitability (action potential firing). During the last decade, an increasing number of studies have described the anomalous expression of VGSC in epithelial cancers where they are associated to motility or invasiveness, therefore increasing the risk of metastasis development. In particular, the neonatal form of Na_*v*_1.5 α-subunit (SCN5A) has been observed in breast cancers where it generates a tonic inward Na^+^ current (in other words a window current) (Roger et al., 2006). It was shown that Na^+^ entry through VGSC leads to the formation and activity of invadopodia with the polymerization of actin and increase in Na^+^-H^+^ exchanger type 1 (NHE1) activity. This mechanism contributes to the acidification of the extracellular surface of the plasma membrane, making a favorable milieu for the activity of acidic cysteine cathepsins (reviewed in Brisson et al., 2013). As previously mentioned, σ1R binds Na_*v*_1.5 with a fourfold symmetry (one σ1R per set of six transmembrane domains). At a more functional level, σ1R silencing in the metastatic breast cancer cell line MDA-MD-231 led to a significant decrease in Na_*v*_1.5 current density, suggesting that the presence of the chaperone in BC cells potentiates their invasive potency (Balasuriya et al., 2012). Interestingly, further studies showed in the same cell line that the functional coupling between σ1R and Na_*v*_1.5 was also involved in cell adhesion properties, but not in cell proliferation or migration (Aydar et al., 2016).

##### hERG and ECM-induced invasive phenotype

Integrin-mediated cell adhesion has a pivotal role on cell fate determination and is intimately associated with ion transport. Ion channels and integrins form signaling hubs that recruits membrane receptors involved in proliferation, migration, differentiation, invasion or angiogenesis. A growing number of studies indicates that ion channels regulate signaling pathways downstream integrin activation by the ECM. Remarkably, their association with integrins determines their open probability, which in turn influences downstream pathways (reviewed in Becchetti et al., 2019). hERG K^+^ channel has been pointed out as a partner of several integrins and is prognostic a marker in several solid tumors and leukemias. As stated above, σ1R binds hERG α-subunits and enhances hERG trafficking to the plasma membrane in K562 leukemia cells or transfected HEK293 cells, leading to increased current density (Crottès et al., 2013; Balasuriya et al., 2014). In myeloid leukemia and CRC, the rapid association of hERG with the β1 subunit of integrin upon ECM stimulation requires σ1R. The silencing of the chaperone abolishes both ECM-induced stimulation of hERG and PI3/AKT pathway downstream of integrin stimulation. Consequently, σ1R inhibition reduces migration, angiogenesis and metastasis spreading *in vitro* and *in vivo* using zebra fish and mice models (Balasuriya et al., 2014; Crottès et al., 2016). These data demonstrate that σ1R dynamically shapes cancer cell electrical signature in response to the tumor microenvironment. The role of σ1R in electrical plasticity within the cancer tissue obviously contributes to the signaling underlying the phenotypic adaptation of cancer cells to a highly stringent environment.

Altogether, these results suggest that the innate pro-adaptive function of σ1R is hijacked to the benefit of tumor development.

## A Unifying Theory

Due to their critical role in regulating trafficking and functions of VGICs, knocking-out classical ion channels’ auxiliary subunits alters a variety of cellular functions (Giese et al., 1998; Pongs et al., 1999; Vacher et al., 2008; Sun et al., 2011; Martinez-Espinosa et al., 2014), which can lead to various chronic diseases such as long QT syndrome (Schulze-Bahr et al., 1997; Splawski et al., 1997), epilepsy (Heilstedt et al., 2001) and even premature death (Arikkath and Campbell, 2003). In contrast, σ1R KO mice are viable and do not exhibit clear behavioral and physiological phenotypes (Langa et al., 2003), raising the hypothesis that σ1R becomes critical only when the system is challenged.

Indeed, σ1R KO mice exhibit behavioral deficits when confronted to novel situations that require behavioral adaptations, such as during learning and memory tasks (Entrena et al., 2009; Chevallier et al., 2011) and during stressful and anxiogenic situations that require coping mechanisms (Sabino et al., 2009; Chevallier et al., 2011). Similarly, in the context of neuronal electrical activity, preventing σ1R activation with various antagonists both in freely moving animals or *in vitro* does not alter Na^+^ currents (Zhang et al., 2009), Ca^2+^ dynamics (Tchedre et al., 2008; Cuevas et al., 2011; Pan et al., 2014) and K^+^ currents (Kourrich et al., 2013). In fact, these findings suggest that σ1R’s functions are not necessary in situations that are not threatening the biological integrity of the individual. This idea is further supported by studies demonstrating that σ1R ligands activation exhibit robust neuroprotective properties, such as reducing infarct volume after embolic stroke (Allahtavakoli and Jarrott, 2011), preventing apoptotic retinal ganglion cell death induced by glutamate and excitotoxic perinatal brain injury (Griesmaier et al., 2012). σ1R ligands also rescue various behavioral and biological deficits associated with amnesia, depression, neuropathic pain, cocaine-induced immune alteration and HIV expression, and Alzheimer’s disease (Maurice and Su, 2009). In stark contrast with these protective properties of σ1R activation, ligand activation of σ1R contributes to tumor growth (Crottès et al., 2013) and the development of psychostimulant addiction (Katz et al., 2011, 2016). Based on these findings, we speculate that primary σ1R’s functions, which are to preserve cellular health and survival, are hijacked to contribute to maladaptive cellular functions, thereby leading to chronic diseases such as cancer and stimulant addiction.

### Stimulant Addiction

Besides their rewarding properties, cocaine and other psychostimulants drugs trigger neurotoxic mechanisms (Pereira et al., 2015). This toxicity originates from various drugs’ mechanisms of actions, such as supraphysiological elevation of extracellular DA. While enhanced DA signaling contributes to the rewarding properties of psychostimulant drugs, it also induces oxidative stress and associated neuronal apoptosis (Pereira et al., 2015). Indeed, increase of oxidative stress in brain regions associated with the brain reward circuits (NAc, frontal cortex, and hippocampus) (Dietrich et al., 2005; Muriach et al., 2010; Jang et al., 2015) upregulates pro-inflammatory mediators (e.g., cytokines and chemokines) or astroglia/microglia activation (Renthal et al., 2009; Piechota et al., 2010; Blanco-Calvo et al., 2014; Lopez-Pedrajas et al., 2015; see review Pereira et al., 2015). Interestingly, a physiologically relevant concentration of DA that does not cause apoptosis becomes toxic in σ1R knockdown cells (Mori et al., 2012), consistent with neuroprotective and other associated positive effects of σ1R ligands activation on various chronic neurodegenerative diseases such as Alzheimer’s (Ryskamp et al., 2019) and Huntington’s diseases (Bol’shakova et al., 2017; Ryskamp et al., 2017; see review Cai et al., 2017). Indeed, σ1R agonist PRE-084 reduces oxidative species, calcium flux and other inflammatory molecules [including interleukin (IL) IL-1β, IL-6, IL-8 and tumor necrosis factor alpha (TNFα)] in various cell types (Katnik et al., 2006; Szabo et al., 2014). As such, we speculate that identified cocaine-driven σ1R mechanism associated with the development of cocaine addiction may be initially a neuroprotective mechanism aimed to counteract deleterious effect of psychostimulant drugs of abuse on neuronal health. Here, we present a hypothesis that may provide clues onto the dual effect of σ1R activation, aimed primarily to be pro-survival but could be hijacked to promote the development of addiction to psychostimulant drugs.

Today, we know that repeated cocaine exposure leads to several long-lasting neuroadaptations in the brain reward circuits (Luscher and Malenka, 2011; Wolf and Tseng, 2012; Kourrich et al., 2015; Wolf, 2016). The mechanisms through which these neuroadaptations are initiated and their contributions to shaping lasting changes in behavior are active fields of research. A recent study provided direct evidence that persistent cocaine-induced neuronal hypoactivity in the NAc shell, now considered as one of the hallmarks for cocaine addiction, is initiated by σ1R activation (Delint-Ramirez et al., 2018). This adapatation of the NAc shell neurons is characterized by a decreased ability for neurons to trigger action potentials and is associated with enhanced hyperpolarizing and decreased depolarizing membrane ion channels. It is tempting to speculate that the primary function of this early σ1R-driven mechanism is to protect the neuron from cocaine-induced cytotoxic mechanisms, a scenario supported by indirect evidence from two lines of studies. Specifically, cocaine administration induces an early increase of membrane NMDA glutamate receptors (NMDARs) in the NAc shell, especially receptors that contain the GluN2B subunit. These GluN2B-containing NMDARs appear to contribute to the development of further lasting changes in glutamate neurotransmission in the reward circuits and thereby may participate to the development of cocaine addiction (Dong and Nestler, 2014). NMDARs are highly permeable to Ca^2+^ and inclusion of GluN2B subunits further prolongs Ca^2+^ entry (Monyer et al., 1994; Vicini et al., 1998; Rumbaugh and Vicini, 1999; Schilstrom et al., 2006). NMDAR-induced Ca^2+^ neurotoxicity is one of the primary factor that leads to cell damage (see review Pchitskaya et al., 2018). A recent study showed that D-cycloserine (DCS), an agonist of NMDARs, leads to decreased neuronal excitability, and disrupting GluN2B prevents both this mechanism and cocaine-induced neuronal hypoactivity in the NAc shell (Wang et al., 2018). Another study found that this same adaptation, i.e., cocaine-induced neuronal hypoactivity, is initiated by direct cocaine binding to σ1R (Delint-Ramirez et al., 2018).

Altogether, cocaine-induced neuronal hypoactivity in the NAc shell may originally be an adaptive mechanism aimed to counteract Ca^2+^-induced neurotoxicity. However, at the circuit level, decreased intrinsic excitability of MSNs, which are inhibitory projection neurons, is expected to disinhibit downstream reward-related brain regions and thereby promote addiction-relevant behaviors (reviewed in Kourrich et al., 2015). Consistent with this framework, Khoshbouei and colleagues found that σ1R activation decreases METH-stimulated DA efflux and prevents METH-induced, DAT-mediated increases in firing activity of DA neurons, which together may also play a role in limiting DA-induced neurotoxicity (Sambo et al., 2017). It is noteworthy to mention that METH is a psychostimulant drug that exhibits strong neurotoxic effects. Importantly, in both cocaine and METH studies, σ1R activation dampens neuronal firing. However, at the behavioral level, resulting effects of σ1R-driven inhibition of neuronal firing in the NAc shell MSNs or ventral tegmental area (VTA) DA neurons, which may be a mechanism limiting activity-dependent neurotoxicity, differentially affect rewarding properties of the drug. While this mechanism supports the role of σ1R in neuroprotection, its effects on the development or maintenance of stimulant abuse likely depends on its brain site of action. Taken together, this theory warrants further studies to elucidate brain region-specific mechanisms by which σ1R, likely depending on client proteins available, contributes to the development of stimulant abuse. This idea is further supported by a recent study demonstrating that cocaine administration induces a cascade of cellular mechanisms in the NAc that aim to counteract each other in homeostatic fashion (Wang et al., 2018), which ultimately heightens cocaine seeking after drug abstinence.

### Cancer

During tumor development, cancer cells are facing intrinsic (oncogene activation) and extrinsic (limiting nutrient or oxygen supply, inflammation) challenges, with which they must cope to survive. Moreover, imbalance between protein folding demand and capacity in the ER leads to a situation of ER stress that is often observed in highly proliferative tumor cells (reviewed in Papaioannou et al., 2018; Obacz et al., 2019). These challenges share a common point with those faced by neurons in many neurodegenerative diseases or stroke for which activation of σ1R revealed beneficial effects (Chu and Ruoho, 2016; Weng et al., 2017; Penke et al., 2018; Smith et al., 2018). The review of studies exploring the function of σ1R in cancers indicates that the protein is generally associated with enhanced invasive properties of cancer cells and a poorer diagnosis at the patient level. In fact, depending on the type of cancer considered, σ1R participates to many hallmark of cancers as defined by Hanahan and Weinberg (2011), including apoptosis resistance, migration, invasive potency, angiogenesis and cell response to the microenvironment. The contribution of σ1R to these hallmarks can be synthesized as follows:

σ1R expression lowers **cell sensitivity to apoptosis** by slowing activation of VRAC channels involved in AVD, but no drastically enough to impede RVD, the latter being required for cell cycle progression (Renaudo et al., 2004, 2007) (Figure 1B).

Enhanced **migration** in BC and CRC cancers is the consequence of a σ1R-dependent formation of the special coupling between a Ca^2+^-dependent channel (SK3) and a Ca^2+^ channel (Orai1) responsible for increased constitutive or capacitive Ca^2+^ entry, which finally triggers cell motility (Guéguinou et al., 2016; Gueguinou et al., 2017). In BC cells, σ1R expression increases Na_*v*_1.5 current density, probably through a direct association between the two proteins (Balasuriya et al., 2012). Since a Na_*v*_1.5-associated Na^+^ window current stimulates NHE1 activity and subsequent extracellular acidification, σ1R likely potentiates **cell invasiveness potency** through increased Na_*v*_1.5 activity (Roger et al., 2003, 2004, 2015; Brisson et al., 2011, 2013) (Figure 1B).

σ1R also deeply influences **the dialog between tumor cells and the microenvironment** by regulating ion channels. By shaping cancer cell electrical signature in response to ECM, σ1R orchestrates the formation of [channel:receptor] complexes at the plasma membrane, contributing to the integration of signals from the tumor microenvironment and the subsequent adaptive phenotype. Indeed, σ1R is a key actor of the formation of [integrin:hERG] signaling hub at the plasma membrane, triggering AKT-dependent pro-metastatic cell behavior *in vivo* by stimulating **migration**, vascular endothelial vascular factor (VEGF) secretion, finally leading to migration, **extravasation** and **angiogenesis** in chronic myeloid leukemia and CRC (Crottès et al., 2016; Becchetti et al., 2019) (Figure 1B).

Altogether, these results support the idea that the innate function of σ1R, aimed at a better cell survival in physiopathological conditions (Hayashi and Su, 2007; Chu and Ruoho, 2016), is hijacked to the benefit of tumor cell growth and invasive properties. It is therefore tempting to postulate that within cancer tissues, σ1R shapes tumor cell electrical signature (Figure 1B), thereby enhancing their adaptation potency to their environment.

## Concluding Remarks

The comprehension of the physiological significance of σ1R as well as the cellular and molecular mechanisms associated with this protein has deeply progressed during the past 10 years. The data accumulated describing the functional interactions between ion channels and σ1R have refined the picture of the contribution of σ1R to CNS diseases including stimulant addiction. The recent data described above demonstrate that contribution to hallmarks of cancer cells and stimulant addiction represent two groups of pathological contexts where [σ1R:ion channel] complexes may play central roles. One of the most striking finding supporting this idea is that a common mechanism, i.e., the chaperoning of voltage-gated K^+^ channels, is involved in both neuronal response to cocaine and cancer cell response to tumor microenvironment. In a unifying view, it is tempting to speculate that both cocaine exposure (Figure 1A) and oncogenic processes (Figure 1B) activate the protective function of σ1R that in turn contributes to a shift in cell homeostasis leading to deleterious behaviors.

## Author Contributions

OS and SK contributed to all sections in the review. OS and SK were entirely responsible for the sections related to cancer and drug addiction, respectively.

## Conflict of Interest

The authors declare that the research was conducted in the absence of any commercial or financial relationships that could be construed as a potential conflict of interest.
